# Analytical Enantio-Separation of Linagliptin in Linagliptin and Metformin HCl Dosage Forms by Applying Two-Level Factorial Design

**DOI:** 10.3390/scipharm84040671

**Published:** 2016-10-17

**Authors:** Sushant B. Jadhav, Rahul M. Mane, Kalyanraman L. Narayanan, Popatrao N. Bhosale

**Affiliations:** 1 Department of Chemistry, Shivaji University, Kolhapur 416-004, Maharashtra, India; jadhavsb@drreddys.com (S.B.J.); maners09@gmail.com (R.M.M.); 2 Research and Development, Integrated Product Development, Dr. Reddy’s Laboratories Ltd., Bachupally, Hyderabad 500-090, Telangana, India

**Keywords:** *S*-isomer, enantiomer, design of experiments (DoE), development, linagliptin and metformin hydrochloride

## Abstract

A novel, stability indicating, reverse phase high-performance liquid chromatography (RP-HPLC) method was developed to determine the *S*-isomer of linagliptin (LGP) in linagliptin and metformin hydrochloride (MET HCl) tablets (LGP–MET HCl) by implementing design of experiment (DoE), i.e., two-level, full factorial design (2^3^ + 3 centre points = 11 experiments) to understand the critical method parameters (CMP) and its relation with the critical method attribute (CMA), and to ensure robustness of the method. The separation of the *S*-isomer, LGP and MET HCl in the presence of their impurities was achieved on Chiralpak^®^ IA-3 (*Amylose tris (3, 5-dimethylphenylcarbamate*), immobilized on 3 µm silica gel) stationary phase (250 × 4.6 mm, 3 µm) using isocratic elution and detector wavelength at 225 nm with a flow rate of 0.5 mL·min^−1^, an injection volume of 10 µL with a sample cooler (5 °C) and column oven temperature of 25 °C. Ethanol:Methanol:Monoethanolamine (EtOH:MeOH:MEA) in the ratio of 60:40:0.2 *v*/*v*/*v* was used as a mobile phase. The developed method was validated in accordance with international council for harmonisation (ICH) guidelines and was applied for the estimation of the *S*-isomer of LGP in LGP–MET HCl tablets. The same method also can be extended for the estimation of the *S*-isomer in LGP dosage forms.

## 1. Introduction

Linagliptin (LGP) is a class of DPP-4 (Dipeptidyl peptidase-4) inhibitor with a unique pharmacokinetic (PK) profile, characterized by negligible renal excretion, and used in the treatment of type II diabetes as glucagon increases blood glucose levels, while DPP-4 inhibitors reduce glucagon and blood glucose levels. The mechanism of DPP-4 inhibitors is to increase incretin levels, GLP-1 and GIP (Glucagon-like peptide-1 and Gastric inhibitory peptide) [1,2,3], which inhibit glucagon release, which in turn increases insulin secretion, decreases gastric emptying, and hence decreases blood glucose levels.

Metformin hydrochloride (MET HCl) is an oral antidiabetic drug in the biguanide class. It is used for the treatment of type II diabetes, particularly, in overweight and obese people and those with normal kidney function. MET works by suppressing gluconeogenesis production by the liver [4,5,6]. Classical insulin secretagogues include sulfonylureas (still recommended because of their low cost) [7], but these glucose-lowering agents implicate a risk of exposure to potentially severe hypoglycaemia [8,9,10,11] weight gain [8,9,11,12,13] and PK interactions [14], which may worsen outcomes [15]. Moreover, LGP and MET HCl are not prone to PK drug–drug interactions. Their co-administration improves blood glucose control more potently than either compound separately, without hypoglycaemia and without increasing metformin related gastrointestinal side effects. Hence, a combination of LGP and MET HCl is very effective to control type II diabetes [16].

To optimise the method parameters, a design of experiments (DoE) study was used. DoE is a rational approach that enables scientists to learn about product/method behaviour by running a series of experiments, where a maximum amount of information is learned, using a minimum number of studies, provided that the chemistry of a given operation is understood. As a result of the DoE study, the method performance can be understood and improved due to knowledge of critical method parameters (CMPs), critical method attributes (CMAs), overlay plots or design space. Hence, a normal operating range (NOR), proven acceptable range (PAR) [17,18] and control strategy, will ensure optimal performance and reliability of the method and the data generated. The most important outcome of a successful DoE based analytical method development is a robust and rugged strategy [17,18,19,20] that will likely be useful for many years with very few limitations; it will also be used for successful regulatory filling and will be adopted in the manufacturing, the analytical laboratory and in quality control departments with strong positive feedback.

There were not any official monographs, or analytical methods were available for the *S*-isomer of LGP–MET HCl tablets of 2.5 mg/500 mg, 2.5 mg/850 mg and 2.5 mg/1000 mg. The label claim of MET HCl (1000 mg) is 400 times higher than LGP (2.5 mg), hence to achieve the limit of quantification (LOQ) of the *S*-isomer, the interference of MET HCl was observed due to a higher test concentration. Present work overcomes such problems by developing a method for the *S*-isomer of LGP–MET HCl tablets of 2.5 mg/500 mg, 2.5 mg/850 mg and 2.5 mg/1000 mg. The same method can be useful for LGP 5 mg tablets as well as different LGP combination drug products.

## 2. Materials and Methods

### 2.1. Chemicals and Reagents

All chemicals, solvents and reagents were of analytical reagent (AR) grade or better, used as received, without further purification for analysis and synthesis. The *S*-isomer, LGP and LGP–MET HCl dosage forms and their impurities (used for specificity) were provided by Dr. Reddy’s Laboratories, (Hyderabad, India). Impurity details (only the LGP, *S*-isomer, MET HCl and Impurity-A are depicted in Table 1, other LGP impurities are not depicted due to intellectual property concern). Ethanol was purchased from Hayman (Witham Essex, UK). High-performance liquid chromatography (HPLC) grade methanol was purchased from Rankem (Thane, India). Monoethanolamine (MEA) was purchased from Loba Chemie (Mumbai, India). Water obtained from Milli-Q water purification system (Merck-Millipore, Bedford, MA, USA) was used for the study.

### 2.2. Equipment and Chromatographic Conditions

Waters and Agilent HPLC with Photo Diode Array (PDA) detector and Empower-2 software (Milford, MA, USA) were used for development and validation. XP2U and XS205 balances (Mettler Toledo, Greifensee, Switzerland) were used for the entire study.

The desired chromatographic separation was achieved on HPLC for the *S*-isomer by using an optimised mixture of EtOH:MeOH:MEA in the ratio of 60:40:0.2 *v*/*v*/*v* as a mobile phase. Chiralpak^®^ IA-3 (*Amylose tris (3, 5-dimethylphenylcarbamate*) immobilized on 3 µm silica gel) column, has a dimension of 250 × 4.6 mm, 3 µm (Daicel, Chiral Technologies Inc., West Chester, PA, USA. (Part No. 80525)). The method employs isocratic elution at 225 nm detector wavelength with a flow rate of 0.5 mL·min^−1^, an injection volume of 10 µL with sample cooler (5 °C) and a column oven temperature of 25 °C has been used.

### 2.3. Preparation of Standard Solution

Standard stock solution was prepared by transferring 4.5 mg of the *S*-isomer dissolved in 170 mL of mobile phase, with the help of a sonicator, into a 200 mL volumetric flask and diluted to the final volume with mobile phase and mixed well. 5.0 mL of the above solution were transferred to a 250 mL volumetric flask and diluted to the final volume with mobile phase and mixed well (Final concentration = 0.45 µg·mL^−1^).

### 2.4. Preparation of Test Solution (LGP–MET HCl-2.5/500 mg, 2.5/850 mg, 2.5/1000 mg)

Twenty tablets of LGP–MET HCl were weighed; the average weight was calculated and then crushed to a fine powder with the help of mortar–pestle and an equivalent of 7.5 mg of LGP fine powder was transferred to a 25 mL volumetric flask (300 μg·mL^−1^). About 15 mL of mobile phase was added and sonicated for about 20 min with intermittent shaking (the sonicator bath temperature was kept between 20 °C and 25 °C) diluted up to the mark with mobile phase and mixed well. A portion of the above solution was centrifuged for about 10 min at 4000 rpm (about 2683× *g*) in a centrifuge machine; the supernatant solution was collected and again centrifuged for 5 min at 4000 rpm (about 2683× *g*) to get clear solution.

## 3. Results

### 3.1. Development and Method optimization

#### Optimization of Column and Mobile Phase

**Figure 1:**
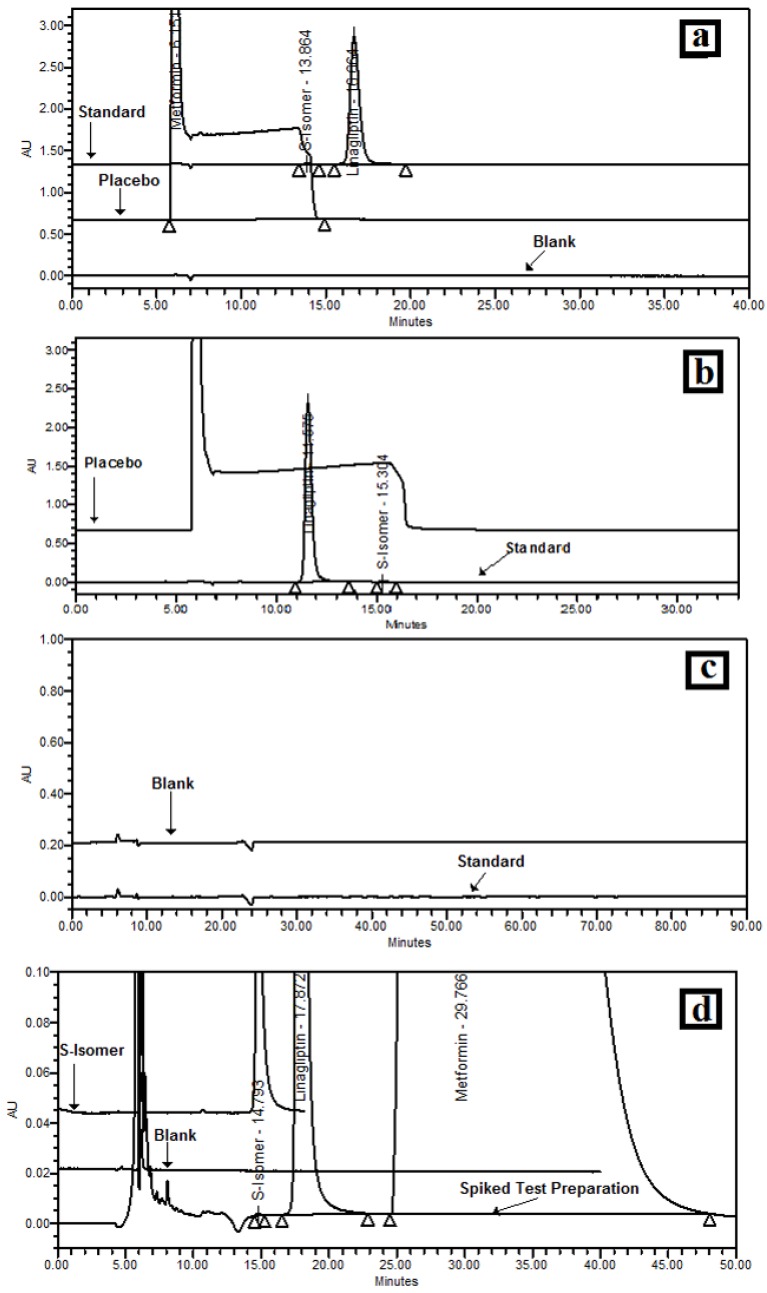
Optimisation of the column and mobile phase (**a**) chromatogram with EtOH:MeOH: monoethanolamine (MEA) (50:50:0.2) *v*/*v*/*v* using Chiralpak AD-H; (**b**) chromatogram with EtOH:isopropanol (50:50) *v*/*v*/*v* using Chiralpak OD-H; (**c**) chromatogram with EtOH:*n*-Hexane (10:90) *v*/*v*/*v* using Chiralpak OD-H; (**d**) chromatogram with EtOH:MeOH:MEA (60:40:0.2) *v*/*v*/*v* using Chiralpak IA-3.

Based on a literature survey, the initial mobile phase was selected as EtOH:MeOH:MEA (50:50:0.2) *v*/*v*/*v*. Methanol was selected as a mobile phase component as LGP is soluble in methanol, and it was decided that the mobile phase would be kept as a diluent to avoid negative peaks. Alcohols were required to obtain a homogeneous solvent mixture as well as for selectivity; hence ethanol was selected as a mobile phase component. MEA was selected as an additive in a mobile phase as LGP is basic in nature (a basic sample requires a basic additive to optimize the chiral separation). Initially, Chiralpak AD-H,column with dimension 250 x 4.6mm, 5 µm was used with flow rate 0.5 mL·min^−1^ and 225 nm wavelength at 25 °C oven temperature. Using these conditions, interference of MET was observed, as shown in Figure 1a. Therefore, the column was changed to Chiralpak OD-H, with a dimension of 250 × 4.6 mm, 5 µm and mobile phase to EtOH:Isopropanol (50:50) *v*/*v*, but, MET interference were also observed, as shown in Figure 1b. No peaks were observed up to 90 min with EtOH:*n*-Hexane (10:90) *v*/*v* as a mobile phase, with Chiralpak OD-H column as shown in Figure 1c. Hence, for better separation of the LGP and *S*-isomer, it was decided to use two Chiralpak IA columns with lower particle size i.e., Chiralpak IA-3, 250 × 4.6 mm, 3 µm (as Chiralpak IA-3 columns allow free choice of any miscible solvents). To compose the mobile phase, EtOH:MeOH:MEA (60:40:0.2) *v*/*v*/*v* were chosen, with flow of 0.5 mL·min^−1^, 225 nm wavelength and 25 °C oven temperature. The *S*-isomer, LGP and Met HCl were well separated from each other but the runtime was about 110 min. Therefore, to reduce the mobile phase, column cost and run time, from 110 min to 60 min, it was decided to use the single column with the same chromatographic conditions as shown in Figure 1d.

### 3.2. Design of Experiments

DoE findings and results obtained by full factorial design are shown in Table 2 while the analysis of variance (ANOVA), shown in Table 3, was obtained by the Design-Expert 9.0.1.0 software (Minneapolis, MN, U.S.).

### 3.3. Resolution between the *S*-isomer and LGP-(***R1***)

**Figure 2:**
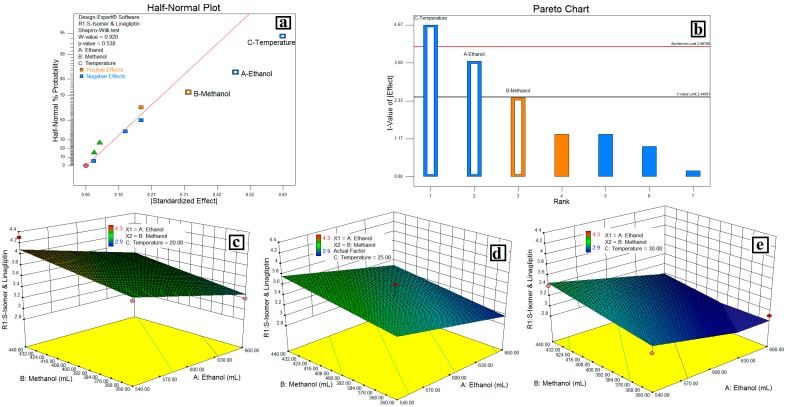
Relation between variables on the resolution of the *S*-isomer and linagliptin (LGP). (**a**) Half Normal Plot; (**b**) Pareto Chart; (**c**, **d** and **e**) 3D Plots showing the linear effect of temperature with volume of EtOH from mobile phase.

**Figure 3:**
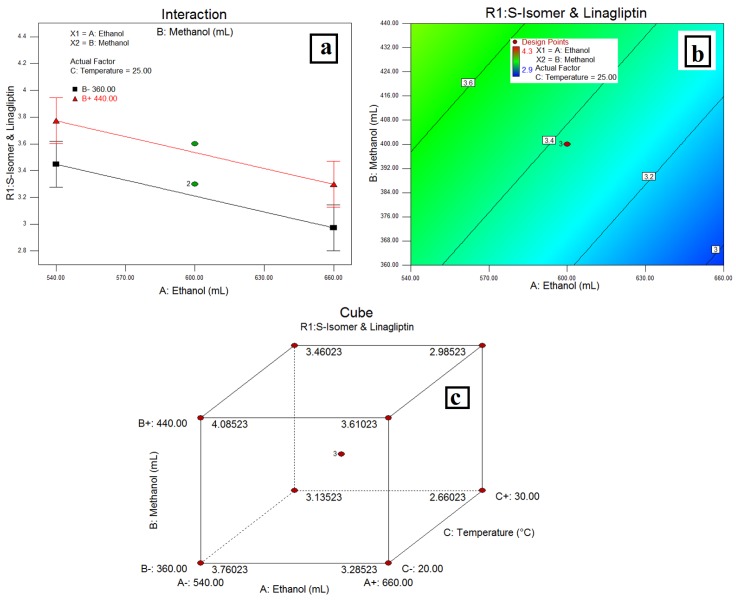
Interaction of MeOH and EtOH on resolution of the *S*-isomer and LGP (**a**); Desirability plot (**b**) and cube (**c**) showing the interaction of EtOH, MeOH and temperature.

The relationship between variables were elicited using half-normal plots, pareto charts, and 3D plots, (Figure 2), while the interaction plot, desirability plot and cube are as depicted in Figure 3.

### 3.4. Resolution between LGP and MET-(***R2***)

**Figure 4:**
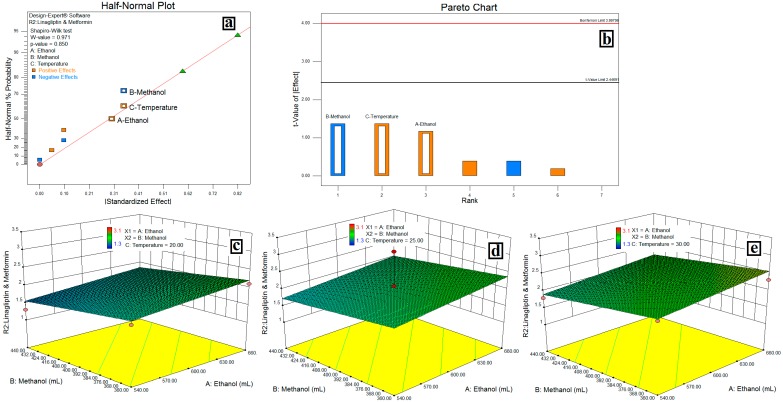
Relation between variables on resolution of LGP and MET HCl (**a**) Half-Normal Plot; (**b**) Pareto Chart; (**c**, **d** and **e**) 3D Plots showing the linear effect of temperature with volume of EtOH from mobile phase.

**Figure 5:**
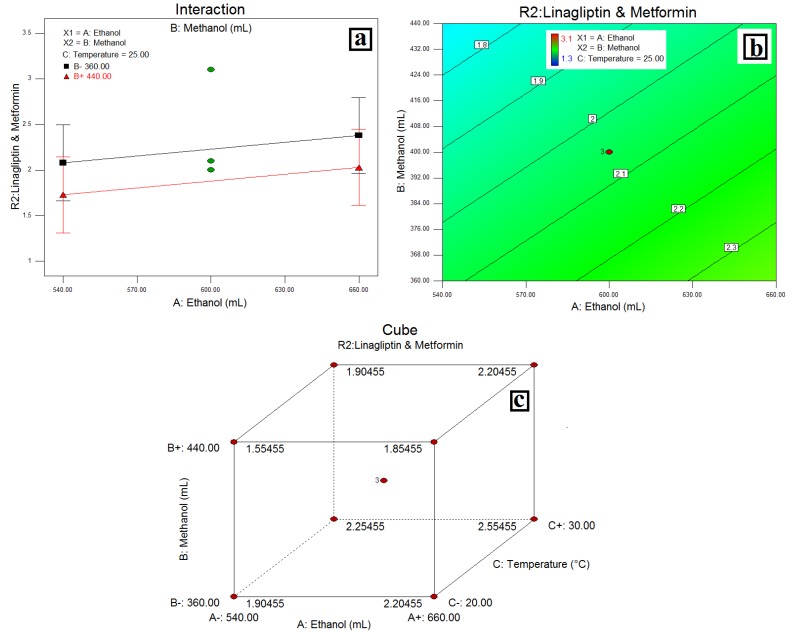
(**a**) Interaction of MeOH and EtOH on resolution of LGP and Met HCl; (**b**) Desirability Plot and (**c**) cube showing the interaction of EtOH, MeOH and temperature.

The relationship between variables was elicited using half-normal plots, pareto charts, and 3D plots, (Figure 4) while the interaction plot, desirability plot and cube are as depicted in Figure 5.

### 3.5. Method Validation

#### 3.5.1. Specificity/Selectivity

**Figure 6:**
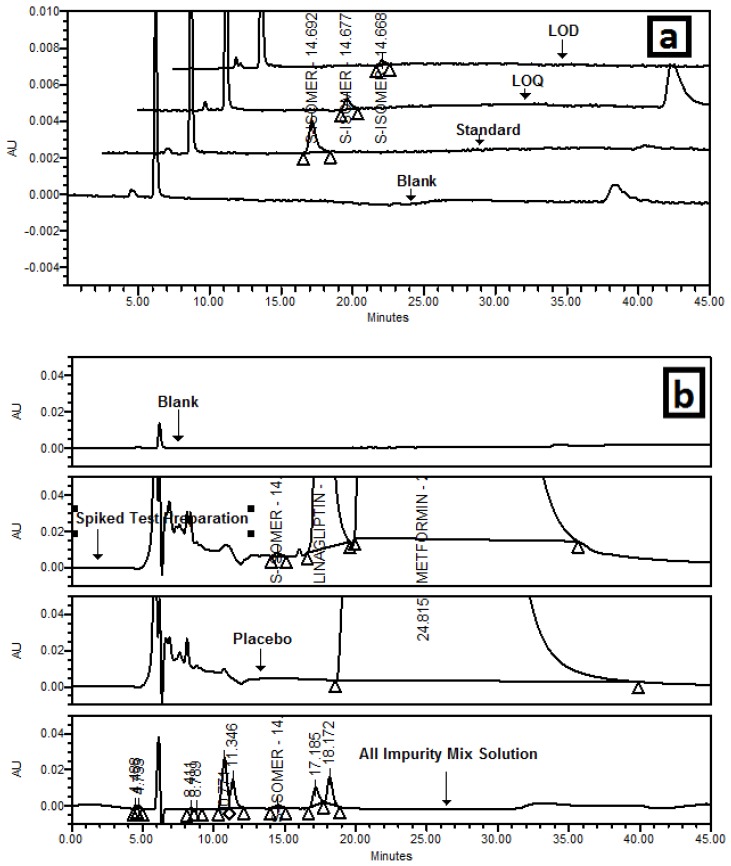
(**a**) Overlaid chromatogram of blank, standard, limit of detection (LOD) and limit of quantitation (LOQ); (**b**) Overlaid chromatogram of a blank, *S*-isomer spiked test preparation, placebo/matrix, LGP and MET HCl impurity mix solution.

It was observed that there was no interference at the retention time (RT) of the *S*-isomer due to a diluent blank, placebo and eight degradation products of the LGP and Met HCl. Chromatograms are shown in Figure 6.

#### 3.5.2. Precision

The precision of the method was demonstrated by injecting six samples, prepared by spiking the test preparation with the *S*-isomer to get a 0.15% concentration of the *S*-isomer; intermediate precision was checked on a different day with a different analyst, column and with a different system. The results of the repeatability and intermediate precision are reported in Table 4. The developed method was found to be precise as the percentage of relative standard deviation RSD was <5%.

#### 3.5.3. Limit of Detection and Limit of Quantitation 

The visual method was adopted to determine the limit of quantification. The quantification limit was achieved by injecting a series of possible dilute solutions of the *S*-isomer. The precision and accuracy at the limit of quantification (LOQ) were also established. The limit of detection (LOD) was 0.015% while the LOQ was 0.046% and the signal to noise ratio was found to be greater than 10. The LOQ precision and accuracy at the LOQ of the *S*-isomer are reported in Table 4 and the percentage of RSD for six preparations is less than 15%.

#### 3.5.4. Accuracy

To confirm the accuracy of the proposed method, recovery experiments were carried out by adding a known amount of the *S*-isomer; corresponding to five concentration levels; 50%, 75%, (*n* = 3) 100% and 200% (*n* = 6) of 0.15% for the *S*-isomer by giving the same treatment as described in the test preparation. Recovery of the *S*-isomer was 100.2% for 50%, 103.0% for 75%, 108.1% for 100% and 106.6% for 200%, with respect to added quantities of the *S*-isomer.

#### 3.5.5. Linearity and Range

The linearity was determined by preparing standard solutions at concentration levels ranging from the LOQ to 200% (0.1359–0.9087 μg·mL^−1^) of 0.15% for the *S*-isomer (*n* = 7); the average peak areas of the *S*-isomer were plotted against corresponding concentrations and obtained the linearity plot. The response was linear as *r* was >0.9989, while the slope was 122,601 and the intercept was 2014.3.

The range was established by confirming that the analytical procedure provides an acceptable degree of linearity, accuracy and precision when applied to samples containing amounts of analyte from the LOQ to 200% of 0.15% for the *S*-isomer.

#### 3.5.6. Robustness

The effect of change in flow rate, +0.2 mL·min^−1^ (i.e., 0.7 mL·min^−1^) was measured. While, low flow (i.e., 0.3 mL·min^−1^) was not performed, as the calibration of HPLC was done up to 0.5 mL·min^−1^, oven temperature (±5 °C), mobile phase organic composition (Methanol ±10%) on the tailing factor, theoretical plates, the % RSD. **R1** and **R2** were studied and tabulated in Table 5. It was confirmed from the system suitability results that the method was robust with respect to variability in the above conditions.

#### 3.5.7. Solution and Mobile Phase Stability

Stability of the test and standard solution was established at refrigerator conditions i.e., 2–8 °C (as diluent contains volatile solvent i.e., ethanol) for 3 days. Test (spiked with *S*-isomer) and standard solutions were re-analyzed after 24 h, 48 h and 72 h time intervals, and the results of percentage of the *S*-isomer was determined and compared against a fresh test and standard solution. The results of test solution and standard solution did not show any significant change after being 3 days at refrigerator conditions (2 to 8 °C); and the similarity factor for the standard solution was in the range of 0.95 to 1.05. The results from solution stability experiments confirmed that the test and standard solutions were stable for up to 3 days at refrigerator conditions.

Stability of the mobile phase was established by storing it at bench top (25°C) for 3 days. The test solution (spiked with *S*-isomer) was analyzed after 24 h, 48 h and after 72 h time intervals and the results of percentage of the *S*-isomer was compared with a fresh mobile phase. The mobile phase did not show any significant change after 3 days at bench top (25 °C). The results from the mobile phase stability experiments confirmed that the mobile phase was stable for up to 3 days at bench top conditions.

#### 3.5.8. Filter Compatibility

Filter compatibility was performed for nylon and polyvinylidene fluoride (PVDF) 0.22 μm syringe filters to confirm the filter compatibility in the proposed analytical method (*n* = 2). The sample was filtered through both syringe filters and the percentage of *S*-isomer was determined and compared with a centrifuged sample. There was no significant change in the percentage of *S*-isomer with respect to the centrifuged sample, which indicates that both nylon and PVDF syringe filters (0.22 µm) have a good compatibility with the sample solution.

## 4. Discussion

### 4.1. Design of Experiments

The main objective of the DoE was to determine the influential variables or factors, the interaction between the variables, where to set the influential factor in order to minimise the effect of uncontrollable factors, and to optimise the method conditions. The column oven temperature and composition of ethanol and methanol in the mobile phase were identified as CMPs by gaining knowledge during the method development and robustness studies. The resolution between the *S*-isomer, LGP and LGP–MET were identified as the CMAs.

There were several types of designs, such as full factorial (2K) and higher order, fractional factorial, Plackett–Burman, and Taguchi design etc. However, full factorial design (with two levels and three variables) was selected as it is able to identify the main effects and factor interactions without any confounding results and it is more useful to optimize 2–4 factors than the other methods.

A factorial model was composed of a list of coefficients multiplied by associated factor levels and this model can be expressed as: Y = β0 + β2 A + β2 B + β3 C + β12AB + β13AC +… where βn is the coefficient associated with the factor n, and the letters, A,B,C… represent the factors in the model. Combinations of factors (such as AB) represent an interaction between the individual factors.

### 4.2. Resolution between the S-Isomer and LGP-(***R1***)

In the half-normal plot, large effects (absolute values) appear in the upper-right section of the plot, i.e., the column oven temperature (C) has a more significant effect on **R1** followed by the volume of EtOH (A) and the volume of MeOH (B) respectively. In the pareto chart, effects above the Bonferroni limit were almost certainly significant, while effects above the *t-value* limit were possibly significant. This indicates that column oven temperature, followed by the volume of EtOH (A) and the volume of MeOH (B) were more significant than other factors on **R1**, the negative effect of temperature and the volume of EtOH. The positive effect of the volume of MeOH from the pareto chart indicates that a decrease in column oven temperature and the volume of EtOH, and an increase in % or volume MeOH increases the resolution between the *S*-isomer and the LGP increases. The same was confirmed with an interaction plot (Figure 3a). Further the 3D plots (Figure 2c–e) show a linear effect of column oven temperature and the volume of EtOH (as a pareto chart) on the resolution between the *S*-isomer and the LGP-(**R1**). Also, a desirability plot and cube (Figure 3b,c) shows that a decrease in column oven temperature and the volume of EtOH will increase the resolution and predicted the resolution of 3.0 between the *S*-isomer and LGP-(**R1**); it was optimum for the applied method conditions.

### 4.3. Resolution between LGP and MET-(***R2***)

In a half-normal plot, the large effects (absolute values)—the volume of MeOH (B) and column oven temperature (C)—have a more significant effect on **R2** followed by %EtOH (A). A pareto chart indicates column oven temperature, followed by the volume of EtOH (A) and the volume of MeOH (B) were more significant than other factors on **R2**, but these effects were below the *t-value* limit and possibly significant. A negative effect of the volume of MeOH and a positive effect of column oven temperature and the volume of EtOH indicates that a decrease in the volume of MeOH and an increase in column oven temperature and the volume of EtOH increases the resolution between LGP and MET increases. The same was confirmed with an interaction plot (Figure 5a). Further, the 3D plots (Figure 4c–e) show a linear effect of column oven temperature and the volume of EtOH (as a pareto chart) on resolution between LGP and MET. Also, a desirability plot and cube (Figure 5b,c) shows that a decrease in the volume of MeOH and an increase in column oven temperature and the volume of EtOH will increase the resolution and predicted the resolution at about 2 between LGP and MET-(**R2**); it was optimum for the applied method conditions.

An ANOVA analysis (Table 3) showing a model *F-value* of 15.45 for response **R1** implies that the model was significant for response **R1**. “*Prob>F*” values were less than 0.1000, indicating that model terms were significant. The “*Lack of Fit F-Value*” was 0.55 and 0.89 for response **R1,** implying that Lack of Fit was not significant and the difference between adjusted and predicted **R2** values were less than 0.2 and *p-value* was less than 0.05, hence the model was significant. It shows a significant effect of column oven temperature and EtOH on resolution **R1**. While the model was not significant for response **R2** i.e., the effect of EtOH, MeOH and column temperature were not significant on response **R2**, the main aim was to separate the *S*-isomer from LGP, MET and their impurities.

**Figure 7:**
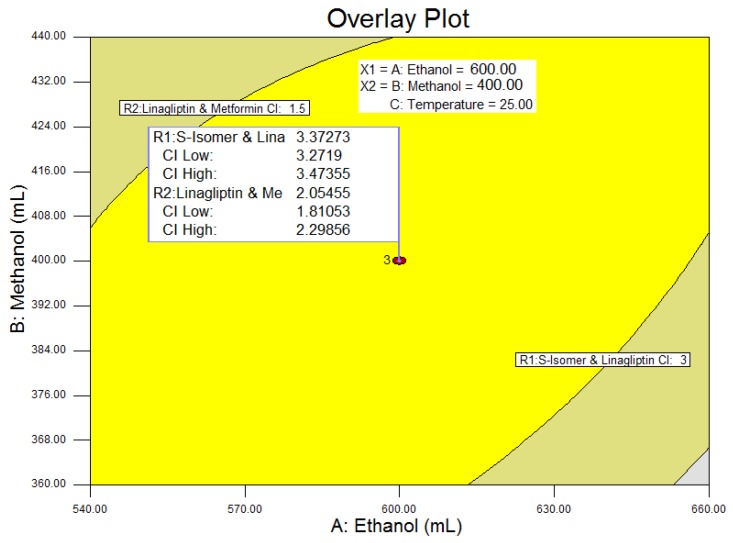
Overlay Plot with confidence interval (CI). Interpretation of the obtained results: ANOVA analysis, desirability and overlay plot. ANOVA: analysis of variance.

An overlay plot (Figure 7) showed that the PAR for the method for column oven temperature was between 20 °C to 28 °C to achieve the resolution greater than 3 and 1.5 for the resolutions **R1** and **R2,** respectively. This indicates that the method was highly sensitive to the change in temperature, especially on resolution **R1,** therefore the NOR for the column oven temperature was ±2 °C. The selected method parameters and chromatographic conditions give the resolutions 3.3 and 2.0 for **R1** and **R2**, respectively, which will ensure minimal problems during quality control analysis. Final chromatographic conditions are as stated in the Equipment and Chromatographic conditions section.

From the method validation data, the test procedure for the *S*-isomer of the LGP in LGP–MET HCl tablets (2.5 mg/500 mg, 2.5 mg/850 mg and 2.5 mg/1000 mg) was found to be linear, precise, accurate, rugged, robust, and specific and indicated stability.

## 5. Conclusions

A new simple, novel and stability indicating reverse phase method was developed by implementing a DoE approach to understand the CMP and its relation with CMA to determine the *S*-isomer of the LGP from LGP–MET HCl pharmaceutical dosage forms. A DoE approach has defined the overlay plot and control strategy to ensure optimum method performance over the lifetime of the product. The method is validated in accordance with ICH guidelines and is found to be accurate, precise, reproducible, robust and specific, which confirms the stability indicating nature of the method. The method is useful for the pharmaceutical industry as LGP has different combination products and is not yet official in any of the pharmacopoeias.

## Figures and Tables

**Table 1:** Chemical structures of linagliptin (LGP) as well as metformin hydrochloride (MET HCl) and their impurities.

Structure	Chemical Name/Molecular Weight/Formula
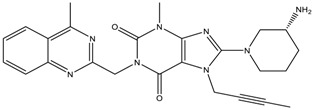	**Linagliptin**: 8-[(3*R*)-3-aminopiperidin-1-yl]-7-(but-2-yn-1-yl)-3-methyl-1-[(4-methylquinazolin-2-yl) methyl]-2, 3, 6, 7-tetrahydro-1H-purine-2,6-dione (472.55) C_25_H_28_N_8_O_2_
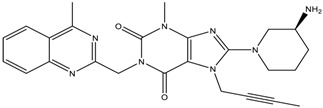	***S*-isomer**: (*S*)-8-3(-aminopiperidin-1-yl]-7-(but-2-yn-1-yl)-3-methyl-1-((4-methylquinazolin-2-yl) methyl)-1H-purine-2, 6 (3H, 7H)-dione (472.55) C_25_H_28_N_8_O_2_
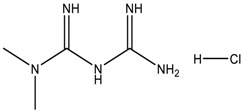	**Metformin HCl**: 1,1-Dimethylbiguanide hydrochloride (165.63) C_4_H_12_ClN_5_
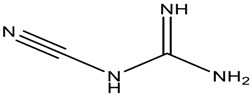	**Impurity-A (MET HCl)**: 1-Cyanoguanidine (84.08) C_2_H_4_N_5_

**Table 2:** Design of experiment (DoE) and results obtained by full factorial design.

			Factor 1	Factor 2	Factor 3	Resolution-1 (R1)	Resolution-2 (R2)
Std.	Run	Type	A: EtOH (%)	B: MeOH (%)	C: Temperature (°C)	*S*-isomer and LGP	LGP and MET
6	1	Factorial	660	360	30	2.9	2.3
4	2	Factorial	660	440	20	3.5	1.8
5	3	Factorial	540	360	30	3.0	2.2
11	4	Center	600	400	25	3.3	2.1
2	5	Factorial	660	360	20	3.2	2.1
9	6	Center	600	400	25	3.3	2.1
7	7	Factorial	540	440	30	3.4	1.8
8	8	Factorial	660	440	30	2.9	2.1
1	9	Factorial	540	360	20	3.7	1.8
3	10	Factorial	540	440	20	4.3	1.3
10	11	Center	600	400	25	3.6	2.0

Std.: standard order; LGP: linagliptin; MET: metformin.

**Table 3:** Analysis of variance (ANOVA) for the selected factorial model, used for prediction and model plots obtained with Design Expert.

Response	Source	Sum of Squares	Degrees of Freedom	Mean Square	*F* Value	*p*-Value Prob > *F*	Model Status	*R*^2^	Adjusted *R*^2^	Predicted *R*^2^
*S*-isomer and LGP	Model	1.44	3	0.48	15.45	0.0018	Significant			
	*A-EtOH %*	*0.45*	*1*	*0.45*	*14.49*	*0.0067*				
	*B-MeOH %*	*0.21*	*1*	*0.21*	*6.78*	*0.0352*				
	*C-Temp. °C*	*0.78*	*1*	*0.78*	*25.08*	*0.0016*				
	Residual	0.22	7	0.031						
	*Lack of Fit*	*0.16*	*5*	*0.032*	*1.05*	*0.5527*	*Not Significant*			
	*Pure Error*	*0.06*	*2*	*0.030*				**0.8688**	**0.8125**	**0.6259**
LGP and MET HCl	Cor total	1.66	10							
	Model	0.67	3	0.22	1.22	0.3700	Not Significant			
	*A-EtOH %*	*0.18*	*1*	*0.18*	*0.99*	*0.3537*				
	*B-MeOH %*	*0.25*	*1*	*0.25*	*1.34*	*0.2846*				
	*C-Temp. °C*	*0.25*	*1*	*0.25*	*1.34*	*0.2846*				
	Residual	1.28	7	0.18						
	*Lack of Fit*	*0.54*	*5*	*0.11*	*0.29*	*0.8852*	*Not Significant*	**0.8781**	**0.8477**	**0.7890**
	*Pure Error*	*0.74*	*2*	*0.37*						
	Cor total	1.95	10							

**Table 4:** Results of method—intermediate and limit of quantification (LOQ) precision and accuracy.

Test Preparation	Method Precision	Intermediate Precision	LOQ Precision	Accuracy at LOQ
1	109.7	109.3	0.053	114.0
2	105.7	108.0	0.043	92.5
3	109.0	106.7	0.042	90.3
4	108.4	112.7	0.045	96.8
5	102.4	110.0	0.046	98.9
6	107.8	114.0	0.049	105.4
**Average**	**107.8**	**110.0**	**0.046**	**99.7**
**Area % RSD**	**3.1**	**2.5**	**8.8**	**NA**

RSD: relative standard deviation.

**Table 5:** Results of robustness.

Variation Parameter	Tailing Factor	Theoretical Plates	Area %RSD	Resolution between *S*-isomer and LGP (R1)	Resolution between LGP and MET (R2)
As per test method	1.3	4511	1.9	3.2	1.6
Flow (0.7 mL/min)	1.4	3698	1.5	3.1	1.5
Temperature: 23 °C	1.1	4675	4.3	3.4	1.0
Temperature: 33 °C	1.4	5280	3.3	2.9	2.0
Methanol: 360 mL	1.4	4467	1.9	2.5	2.7
Methanol: 440 mL	1.4	4696	2.5	3.4	1.2
